# Role of Chemokine Cxcl12a in Mediating the Stimulatory Effects of Ethanol on Embryonic Development of Subpopulations of Hypocretin/Orexin Neurons and Their Projections

**DOI:** 10.3390/cells12101399

**Published:** 2023-05-16

**Authors:** Nushrat Yasmin, Adam D. Collier, Abdul R. Abdulai, Olga Karatayev, Boyi Yu, Milisia Fam, Sarah F. Leibowitz

**Affiliations:** Laboratory of Behavioral Neurobiology, The Rockefeller University, New York, NY 10065, USA

**Keywords:** chemokine, Cxcl12, hypocretin, orexin, ethanol, prenatal alcohol exposure, zebrafish

## Abstract

Studies in zebrafish and rats show that embryonic ethanol exposure at low-moderate concentrations stimulates hypothalamic neurons expressing hypocretin/orexin (Hcrt) that promote alcohol consumption, effects possibly involving the chemokine Cxcl12 and its receptor Cxcr4. Our recent studies in zebrafish of Hcrt neurons in the anterior hypothalamus (AH) demonstrate that ethanol exposure has anatomically specific effects on Hcrt subpopulations, increasing their number in the anterior AH (aAH) but not posterior AH (pAH), and causes the most anterior aAH neurons to become ectopically expressed further anterior in the preoptic area (POA). Using tools of genetic overexpression and knockdown, our goal here was to determine whether Cxcl12a has an important function in mediating the specific effects of ethanol on these Hcrt subpopulations and their projections. The results demonstrate that the overexpression of Cxcl12a has stimulatory effects similar to ethanol on the number of aAH and ectopic POA Hcrt neurons and the long anterior projections from ectopic POA neurons and posterior projections from pAH neurons. They also demonstrate that knockdown of Cxcl12a blocks these effects of ethanol on the Hcrt subpopulations and projections, providing evidence supporting a direct role of this specific chemokine in mediating ethanol’s stimulatory effects on embryonic development of the Hcrt system.

## 1. Introduction 

Exposure to ethanol in utero causes long-lasting neuronal changes in the offspring [1,2]. Studies in rodent models show that prenatal exposure to ethanol at low-moderate concentrations stimulates neuronal development, increasing the proliferation, neurogenesis and migration of neurons expressing the hypothalamic peptide, hypocretin/orexin (Hcrt) [3,4,5,6]. This stimulatory effect on peptidergic neurons is shown in rats to be anatomically specific, with ethanol increasing the number of enkephalin-expressing neurons in the core but not shell of the nucleus accumbens (NAc) [5] and also causing Hcrt neurons to be ectopically expressed outside the hypothalamus, further anterior in the NAc core and ventromedial caudate putamen [7]. 

These findings in rodents have been confirmed and further extended in zebrafish, an especially genetically tractable model that is useful for neurodevelopmental studies due to their transparency and small size, permitting observance of the embryonic brain in vivo and enabling experiments to be performed that are difficult or impossible to achieve using rodent models [8]. In zebrafish embryos and larvae, embryonic ethanol exposure at a low-moderate dose (0.5% *v*/*v*) in the water increases the number of Hcrt neurons in the anterior hypothalamus (AH) in an area-dependent manner, specifically in the most anterior (aAH) but not posterior (pAH) region of the AH [9,10]. Ethanol also causes some of these aAH neurons in zebrafish to migrate further anterior and to be ectopically expressed in the preoptic area (POA) [7,11], consistent with other zebrafish studies showing ethanol inducing ectopically located oxytocin neurons and facial branchial motor neurons [12,13]. The induction of these ectopic neurons is likely due to ethanol’s effects on the timing and directionality of neuronal migration [4,12,14], resulting in their clustering at inappropriate sites that leads to the formation of heterotopias [15], a phenomenon observed in the brains of children with fetal alcohol spectrum disorders [16].

In addition to affecting the number and location of neurons, embryonic exposure to ethanol also alters axonal pathfinding and the morphology of neuronal projections [17,18]. These effects are also brain-area dependent, with ethanol increasing the number, length and complexity of projections from neurons in the hippocampus [19] while decreasing these measures from neurons in the prefrontal cortex [20,21]. We recently found in both rats and zebrafish that ethanol increases the number of processes emanating from the soma of normally located Hcrt neurons [7]. Further, while having little effect on the short projections from Hcrt neurons to local regions, ethanol in zebrafish stimulates the long descending projections from pAH Hcrt neurons that innervate the locus coeruleus (LC) and the long ascending projections from aAH Hcrt neurons that become ectopically expressed in the POA and innervate the subpallium (SP) and dorsal pallium (DP) [9]. 

Studies suggest that the neuroimmune system, including neuronal chemokines, may be involved in these ethanol-induced changes in the development of Hcrt neurons and their projections [10,22,23]. The chemokine ligand, Cxcl12, and its receptor, Cxcr4, are shown to stimulate the proliferation and migration of neurons [24,25] and have a role in axonal guidance and dendritic development [26,27,28,29]. Additionally, the homologues Cxcl12a and Cxcr4b in zebrafish are found to colocalize with Hcrt neurons under control conditions, with ethanol increasing their expression in the hypothalamus and their colocalization with Hcrt neurons in embryos, and these effects are blocked by pretreatment with a Cxcr4 receptor antagonist prior to ethanol exposure [10,22].

Here we sought to determine the underlying neuroimmune mechanisms of ethanol’s anatomically specific effects on the Hcrt system. Specifically, using genetic overexpression and knockdown of the *cxcl12a* gene followed by live imaging of the Hcrt system in larval *Hcrt:EGFP* transgenic zebrafish, we investigated the direct role of the chemokine Cxcl12a in mediating the stimulatory effects identified in zebrafish of embryonic ethanol exposure on the development of anatomically localized and ectopic Hcrt neuronal subpopulations and their projections. 

## 2. Materials and Methods

### 2.1. Animals and Housing

Transgenic *Hcrt:EGFP* [30] zebrafish (*Danio rerio*) of the AB strain background were used in this study. Adult zebrafish were group-housed in 3 L tanks (Aquatic Habitat, Apopka, FL, USA), with recirculating water flow at a temperature between 28 and 29 °C and a pH between 6.9 and 7.4 as previously described [11]. All breeding and raising of embryos occurred within an AAALAC-accredited facility using protocols approved by the Rockefeller University Institutional Animal Care and Use Committee and guidance of the NIH Guide for the Care and Use of Laboratory Animals. This study was carried out in compliance with the ARRIVE guidelines.

### 2.2. Embryonic Ethanol Treatment 

Embryonic exposure of zebrafish to ethanol was performed, as described in our previous reports [11,22] and briefly summarized here. At 22 h post-fertilization (hpf), embryos were removed from an incubator and placed in a solution of either 0.0% (control) or 0.5% (vol/vol%) ethanol for 2 h, washed in fresh embryo medium, and then returned to the incubator. This timing of ethanol exposure from 22 to 24 hpf was selected because it coincides with the beginning of the development of the zebrafish hypothalamus and is when Hcrt neurons begin migrating [10].

### 2.3. cxcl12a mRNA Synthesis and Injection

*Cxcl12a* plasmid [31] with a complete coding sequence subcloned into a pGEM-T vector (provided by Dr. Yoshihiro Yoshihara lab, Riken Center for Brain Science, Japan) was linearized with NcoI restriction enzyme (ThermoFisher Scientific, Waltham, MA, USA). Then, a mMESSAGE mMACHINE T7 Transcription kit (ThermoFisher Scientific, MA, USA) was used to generate capped *cxcl12a* mRNA transcripts, according to the manufacturer’s protocol. A mixture of MO^sp^ (850 ng/µL) and *cxcl12a* mRNA (50 ng/µL) diluted in a 0.1 M KCl vehicle solution was used for rescue experiments. This dose of *cxcl12a* mRNA was chosen based on initial tests showing it to have no effect on morphology or survival, in contrast to higher concentrations (100 ng/µL, 150 ng/µL and 200 ng/µL) which were found to reduce embryo survival. For negative control experiments, both control and ethanol-exposed fish were injected with the vehicle solution. Injections were performed using a Pneumatic PicoPump Micro-injector (World Precision Instruments, Sarasota, FL, USA). The groups tested in experiments involving *cxcl12a* mRNA injection compared to ethanol consisted of (1) control zebrafish injected with vehicle (“Control + Vehicle”); (2) ethanol-exposed zebrafish injected with vehicle (“Ethanol + Vehicle”); and (3) control zebrafish injected with *cxcl12a* mRNA (“Control + *cxcl12a* mRNA”).

### 2.4. cxcl12a Morpholino Design and Injection

To achieve knockdown of Cxcl12a protein expression, we designed a splice morpholino (MO) (MO^sp^: 5′-AAGTGCAGATACTCACATGACTTGG-3′) which was synthesized by Gene Tools (Philomath, OR, USA) and analyzed with the NCBI BLAST tool (https://blast.ncbi.nlm.nih.gov/Blast.cgi, accessed on 15 December 2022) to assess any potential off-target effects. The MO^sp^ was designed to target *cxcl12a* pre-mRNA splicing at exon 2/intron 2 (E2I2) and bind to the last 9 bases of E2 and first 12 bases of I2. This effectively skips exon 2 entirely with its 118 bp. Moreover, we additionally used a Cxcl12a translation blocking MO (MO^aug^: 5′-TTGAGATCCATGTTTGCAGTGTGAA-3′) [28,32] to validate that, unlike the MO^sp^, it does not create alternate transcripts to decrease Cxcl12a mRNA levels. Two doses of MO^sp^, including a low dose of 85 ng/µL and a high dose of 850 ng/µL, were tested to determine the highest and most effective concentration that does not induce a phenotype in wild-type embryos. An amount of 1 nl of both the MO^sp^ and a standard control MO (5’-CCTCTTACCTCAGTTACAATTTATA-3’) at 1 mM [33] were injected into single-cell stage *Hcrt:EGFP* embryos. We determined that the 850 ng/µL of MO^sp^ effectively induced alternate splicing and then used a translation tool from www.ExPASy.com to determine the amino acids produced by alternately spliced *cxcl12a* mRNA. No morphological abnormalities were observed in the injected zebrafish at both high and low doses. The groups tested in experiments involving the *cxcl12a* MO^sp^ consisted of (1) control zebrafish injected with the control MO (“Control + Control MO”); (2) ethanol-exposed zebrafish injected with the control MO (“Ethanol + Control MO”); (3) ethanol-exposed zebrafish injected with the *cxcl12a* MO^sp^ (“Ethanol + *cxcl12a* MO^sp^”); and (4) control zebrafish injected with the *cxcl12a* MO^sp^ (“Control + *cxcl12a* MO^sp^”).

### 2.5. RNA Extraction, cDNA Synthesis and RT-PCR

To confirm the splicing efficiency of the *cxcl12a* MO^sp^, total RNA was extracted from the whole embryo at 28 hpf using TRIzol reagent (Invitrogen, Carlsbad, CA, USA) [34]. An amount of 1 μg of total RNA was used to synthesize cDNA, according to the manufacturer’s procedure in the TaqMan Reverse Transcription kit (ThermoFisher Scientific, Waltham, MA, USA). Using the Phire Tissue Direct RT-PCR Kit (ThermoFisher Scientific, Waltham, MA, USA) and the primers (F: 5′-GTAGTCGCTCTGATGGCGGT-3′ and R: 5′-AGTCCTCTGGCATGCTTGCT-3′), we amplified a region that partially spans exons 1 and 4 and the full length of exons 2 and 3 of the mature *cxcl12a* transcript. The RT-PCR reaction was run at a 65.5 °C annealing temperature in 30 cycles. 

### 2.6. Microscopy and Image Analysis 

To acquire images of Hcrt neurons and their projections throughout the brain, transgenic 6 dpf *Hcrt:EGFP* zebrafish were live-imaged using confocal microscopy, with a 25× objective lens on a Zeiss LSM 780 laser scanning confocal microscope and a z step of 1.0 μm. All images were analyzed using Imaris 9.9.1 software. The AH of the zebrafish brain was first divided into equal halves along the AP axes, into regions defined as anterior AH (aAH) and posterior AH (pAH). The number of Hcrt neurons in the aAH and pAH and also further anterior in the POA was quantified manually using the “Spots” function in Imaris. We then created 3D reconstructions of neuronal projections from Hcrt neurons located in the aAH, POA and pAH using the “Filaments” function of Imaris [35]. Spot sizes for the start point and seeding points of the filament were 7 µm and 0.9 µm with a threshold of 1350 and 350, respectively, followed by manual correction. To quantify the density of terminal points and branch points within certain brain areas of interest, we first used the “Surface” function of Imaris to mask out functionally relevant brain regions that are known to be innervated by Hcrt neurons, as described [36,37]. These included the subpallium (SP), dorsal pallium (DP), locus coeruleus (LC) and lateral forebrain bundle (LFB). For this analysis, we excluded the Hcrt projections to the furthest anterior regions including the olfactory bulb, pineal gland and optic tectum and those to the most posterior regions beyond the LC including the spinal cord. The anatomy for each brain region was derived from the Zebrafish Brain Browser atlas, following previously published guidelines [38]. The density of branch points and terminal points was calculated within each of these brain areas by quantifying their number and then dividing these values by the volume (exported from Imaris “Surface” function) of each respective region. 

### 2.7. Statistical Analyses 

All number data for Hcrt neurons in the aAH and pAH were analyzed using two-way ANOVA, which tested the main effects of the condition and brain area and their interaction, followed by post-hoc Holm–Sidak’s multiple comparisons test. The projection data of Hcrt neurons derived from the brain regions innervated by these neurons was analyzed by a one-way ANOVA for each individual brain area. With the ectopic POA neurons and their projections absent in the control conditions, the POA data from the experimental groups are presented in the text of Section 3 but could not be statistically compared to a control group as there were no POA neurons present in the control zebrafish. All tests were two-tailed, and significance was determined at *p* < 0.05. All data were analyzed using Prism (version 9, GraphPad, San Diego, CA, USA) and are presented as mean ± SEM in the figures and tables.

## 3. Results 

### 3.1. Confirmation of cxcl12a mRNA and MO^sp^

**Figure 1:**
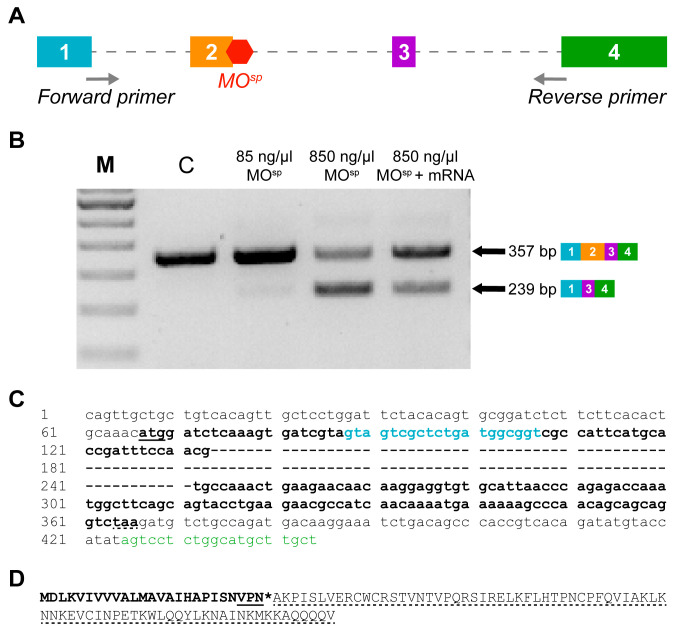
Verification of the efficiency of injection of a splice-altering *cxcl12a* MO (MO^sp^) and of the rescue with *cxcl12a* mRNA in zebrafish embryo. (**A**) A schematic representation of *cxcl12a* pre-mRNA showing its 4 exons, introns (dotted line) and the binding position of MO^sp^ on exon 2 to cause alternate splicing. The positions of the MO^sp^ detection primers (designed to obtain product smaller than full-length gene of 1521 bp), which partially spans exons 1 and 4 and the full length of exons 2 and 3, are shown. (**B**) RT-PCR analysis of Cxcl12a transcript confirming the splice-altering effect of MO^sp^ (850 ng/µL) compared to embryos injected with control MO. A low dose (85 ng/µL) MO^sp^ has no marked splice-altering effect, whereas MO^sp^ + mRNA shows rescue of wild-type (WT) product and increases its expression. M is a 100 bp ladder. A smaller PCR product (239 bp) was observed from MO^sp^-injected embryos because exon 2 (118 bp) was completely skipped; the WT product was 357 bp. (**C**) A segment of the *cxcl12a* gene is shown. The underline and dotted underline show the start and stop codons, respectively. The nucleotides in bold show the coding sequence (cds), whereas the hyphens show the 118 nucleotides of exon 2 that were excised after MO^sp^ injection. Finally, the colored nucleotides show the positions of the forward primer (blue) on exon 1 and the reverse primer (green) on exon 4. (**D**) The resulting truncated transcript, which has 77 amino acids removed (dotted underline), is expected to be only 25 amino acids long (bold) and has 3 new insertions (underline). The asterisk shows the premature stop codon.

To assess the function of Cxcl12a in mediating the ethanol-induced effects on Hcrt neurons and their projections, we used in vitro-transcribed *cxcl12a* mRNA and an antisense splice-altering MO (MO^sp^) injected at the single-cell stage. Injection of the *cxcl12a* mRNA was to overexpress Cxcl12a protein, whereas the MO^sp^ was used to knock down the cxcl12a gene by inhibiting the splicing of exon 2 into the mature mRNA product. The *cxcl12a* gene, located on chromosome 13, generates a 4-exon transcript that encodes a protein composed of 99 amino acids. The schematic in Figure 1A shows the sites on the *cxcl12a* pre-mRNA to which the MO^sp^ binds. Whereas the low dose (85 ng/µL) MO^sp^ has no splice-altering effect, targeted binding of the high dose (850 ng/µL) MO^sp^ causes alternate splicing that fully excludes exon 2 from the mature *cxcl12a* mRNA (Figure 1B). The removal of exon 2 creates a smaller PCR product (239 bp), which can cause translation failure and therefore effectively knock down Cxcl12a protein expression [39,40].

This decreased expression is detected by RT-PCR, which also shows that rescue by co-injecting *cxcl12a* MO^sp^ and *cxcl12a* mRNA increases the expression of the wild-type band intensity compared to the mutant band intensity (Figure 1B). Moreover, sequence analysis shows complete deletion of the 118 bp of exon 2 (Figure 1C), and from this analysis, the resulting transcript encoding a 25 amino acid protein with a premature stop codon is expected (Figure 1D). Taken together, the MO^sp^ effectively truncates the mature *cxcl12a* mRNA, which causes a frameshift in the downstream sequence to insert a premature termination codon and delete 77 amino acids that ultimately decreases wild-type Cxcl12a expression.

### 3.2. Cxcl12a Overexpression Mimics the Ethanol-Induced Increase in Number of Hcrt Neurons in the aAH and POA with No Effect in the pAH

**Figure 2:**
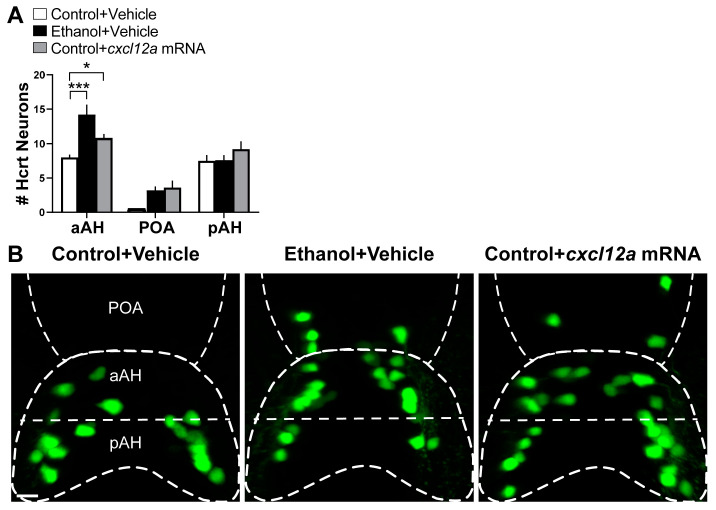
Effects of exposure to embryonic ethanol (0.5% *v*/*v*, 22–24 hpf) and injection of *cxcl12a* mRNA (50 ng/µL) on the number of Hcrt neurons in the aAH, POA and pAH of 6 dpf *Hcrt:EGFP* zebrafish. (**A**) Bar graphs (n = 4–5/group) show the number of Hcrt neurons in the aAH, POA and pAH (Two-way ANOVA, Condition main effect: (F (2, 33) = 8.577, *p* = 0.0009), Brain area main effect: (F (2, 33) = 68.52, *p* < 0.0001), Condition ×Brain area interaction: (F (4, 33) = 3.431, *p* = 0.019), followed by Holm–Sidak post-hoc analysis). (**B**) Photomicrographs (25×, dorsal view) illustrate Hcrt neurons (green) that are located in the aAH, POA and pAH of Control + Vehicle (left), Ethanol + Vehicle (middle) and Control + *cxcl12a* mRNA (right) zebrafish. Scale bar: 10 µm. All results are shown as means ± standard errors. * *p* < 0.05, *** *p* < 0.001. Abbreviations: aAH: anterior part of the anterior hypothalamus, POA: preoptic area, pAH: posterior part of the anterior hypothalamus, Hcrt: hypocretin, hpf: hours post fertilization, dpf: days post fertilization.

We first tested in 6 dpf zebrafish if the overexpression of Cxcl12a has effects similar to those produced by embryonic ethanol exposure (0.5% *v*/*v*, 22–24 hpf), specifically on the number of Hcrt neurons in the aAH, POA and pAH subpopulations. Analysis of *Hcrt:EGFP* zebrafish showed that the Hcrt neurons, while similar in number in the aAH and pAH and not evident in the POA of the Control + Vehicle-injected zebrafish, were significantly altered in the Ethanol + Vehicle-injected zebrafish (Figure 2A), as illustrated in the photomicrographs (Figure 2B). Ethanol increased the number of Hcrt neurons specifically in the aAH (*p* = 0.0001), and it additionally caused the most anterior Hcrt neurons of the aAH (3.2 ± 0.583) in Control + Vehicle zebrafish to become ectopically expressed further anterior in the POA, while having no effect on the number of Hcrt neurons in the pAH (*p* = 0.939). Notably, injection of *cxcl12a* mRNA in the Control + *cxcl12a* mRNA zebrafish had very similar effects to those of embryonic ethanol exposure (Figure 2A,B). It produced a significant increase in the number of Hcrt neurons in the aAH (*p* = 0.019) and again caused some (3.6 ± 1.03) of the most anterior aAH Hcrt neurons to become ectopically expressed in the POA. It also had no effect on the number of Hcrt neurons in the pAH (*p* = 0.497). These results demonstrate that *cxcl12a* overexpression has the same effects as embryonic ethanol exposure on the development of these Hcrt subpopulations.

### 3.3. Cxcl12a Overexpression Mimics the Ethanol-Induced Stimulation of Hcrt Projections to Distant Areas in the Anterior and Posterior Brain

**Figure 3:**
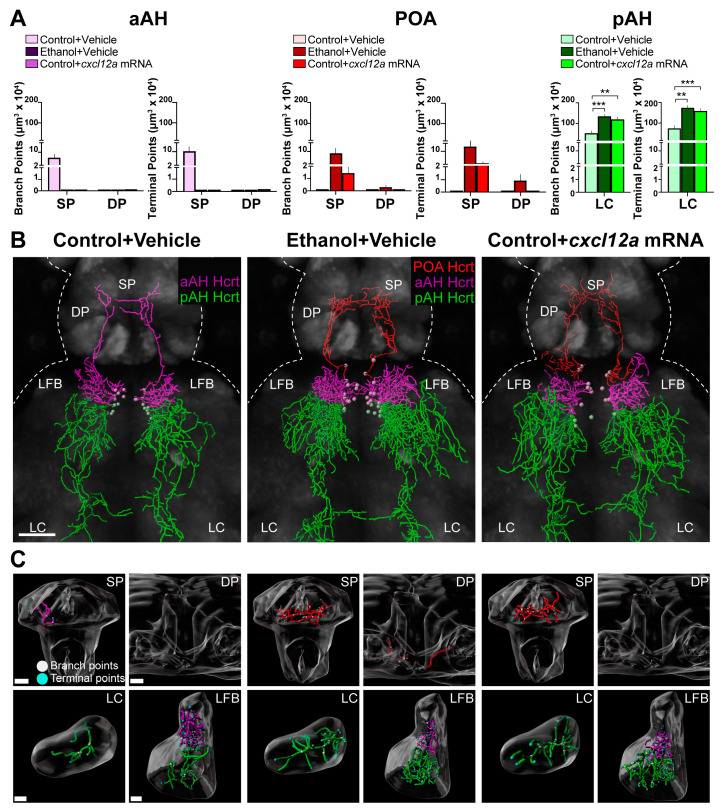
Effects of exposure to embryonic ethanol (0.5% *v*/*v*, 22–24 hpf) and injection of *cxcl12a* mRNA (50 ng/µL) on the density of branch points and terminal points in different brain areas of the projections from Hcrt neurons in the aAH, POA and pAH subpopulations of 6 dpf *Hcrt:EGFP* zebrafish. (**A**) Bar graphs (n = 4–5/group) with purple-colored bars show projection data of aAH Hcrt neurons and their branch points (left) and terminal points (right) in the SP and DP. Bar graphs (n = 4–5/group) with red-colored bars show projection data of ectopic POA Hcrt neurons and their branch points (left) and terminal points (right) in the SP and DP. Bar graphs (n = 4–5/group) with green-colored bars show projection data of pAH Hcrt neurons and their branch points (left, F (2, 10) = 17.14, *p* = 0.0006) and terminal points (right, F (2, 10) = 15.91, *p* = 0.008) in the LC. (**B**) Images show digital representations of Hcrt neuronal projections, created from photomicrographs (25×, dorsal view) of the Control + Vehicle-injected (left), Ethanol + Vehicle-injected (middle) and Control + *cxcl12a* mRNA-injected (right) zebrafish, obtained using confocal microscopy with the “Filaments” function of Imaris software. As with the bar graph data, the primarily ascending aAH Hcrt projections are shown in purple, the ascending POA Hcrt projections are shown in red and the primarily descending pAH Hcrt projections are shown in green. (**C**) Digitally constructed enlargements of the projections in the SP, DP, LC and LFB from the aAH (purple), POA (red) and pAH (green) Hcrt neurons are shown below, with branch points indicated by white dots and terminal points indicated by blue dots. Scale bars: low magnification 50 µm; SP: 15 µm; DP: 15 µm; LC: 10 µm; LFB: 10 µm. All results are shown as means ± standard errors. ** *p* < 0.01, *** *p* < 0.001. Abbreviations: aAH: anterior part of the anterior hypothalamus, POA: preoptic area, pAH: posterior part of the anterior hypothalamus, LFB: lateral forebrain bundle, SP: subpallium, DP: dorsal pallium, LC: locus coeruleus, Hcrt: hypocretin, hpf: hours post fertilization, dpf: days post fertilization.

Here we tested whether the overexpression of Cxcl12a has similar effects to those produced by embryonic ethanol exposure (0.5% *v*/*v*, 22–24 hpf) on the projections of the aAH, POA and pAH subpopulations. As previously described [9], we examined the long projections from the aAH Hcrt neurons that project anteriorly to the SP and DP and are not seen in any posterior structures, the ectopic POA Hcrt neurons that, like aAH Hcrt neurons, project anteriorly to the SP and DP, and the pAH Hcrt neurons that project posteriorly to the LC and are not seen in any anterior structures. We also examined the short projections from the aAH and pAH neurons that project laterally to the LFB. While the branch points (6.6 ± 2.08) and terminal points (10.20 ± 2.54) of the long projections from aAH Hcrt neurons in the Control + Vehicle zebrafish were evident in the SP but not the DP, these projections were not detected in either of these areas from aAH Hcrt neurons in the Ethanol + Vehicle or the Control + *cxcl12a* mRNA zebrafish (Figure 3A), as illustrated in the photomicrographs (Figure 3B) and brain region enlargements (Figure 3C). This is due to the loss in these two experimental groups of the specific Hcrt neurons which, while originally in the most anterior region of the aAH with long projections ascending to the SP, were induced by ethanol and Cxcl12a mRNA to migrate further anterior to become ectopically expressed in the POA. The branch points and terminal points of the projections from these ectopic POA neurons in the Ethanol + Vehicle zebrafish were detected not only in the SP (9.16 ± 2.76 and 12.80 ± 3.05, respectively) but also more dorsally in the DP (0.30 ± 0.19 and 0.88 ± 0.46), an area not reached by the projections from the aAH neurons in Control + Vehicle zebrafish. In the Control + *cxcl12a* mRNA zebrafish, they were similarly detected in the SP (1.44 ± 0.52 and 2.64 ± 0.87, respectively), although they were not evident in the DP (Figure 3A–C). Like these long anterior projections from the POA Hcrt neurons, ethanol increased the branch points (*p* = 0.0004) and terminal points (*p* = 0.00046) of the long projections from the pAH Hcrt neurons in the LC, and *cxcl12a* mRNA injection had a similar effect on these measures of the pAH projections to the LC (*p* = 0.003 and *p* = 0.002, respectively). In contrast to these long projections, both ethanol and *cxcl12a* mRNA had no effect on the branch points and terminal points of the short, lateral projections to the LFB from the aAH and pAH subpopulations of Hcrt neurons (Table 1). These results demonstrate that overexpression of Cxcl12a produces the same effects as embryonic ethanol exposure on Hcrt neuronal projections, indicating that Cxcl12a is an important mediator of ethanol’s effects on Hcrt development. 

### 3.4. Cxcl12a Knockdown Blocks the Ethanol-Induced increase in Number of Hcrt Neurons in the aAH and POA

**Figure 4:**
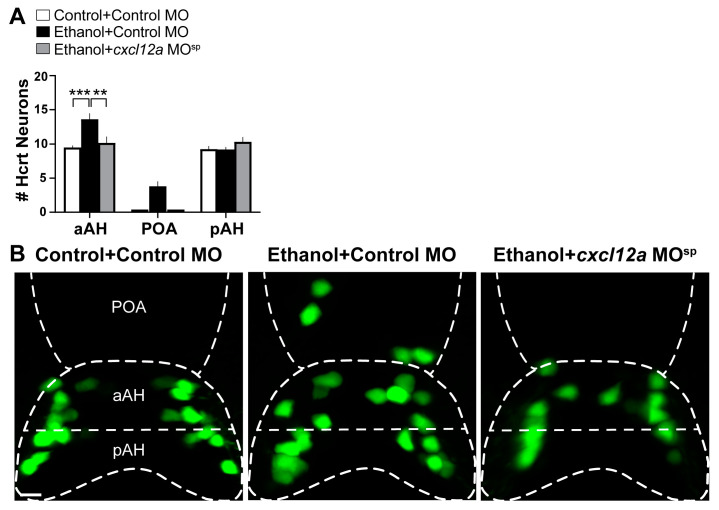
Effects of exposure to embryonic ethanol (0.5% *v*/*v*, 22–24 hpf) and injection of *cxcl12a* MO^sp^ (850 ng/µL) on the number of Hcrt neurons in the aAH, POA and pAH subpopulations of 6 dpf transgenic *Hcrt:EGFP* zebrafish brains. (**A**) Bar graphs (n = 4–6/group) show the number of Hcrt neurons in the aAH, POA and pAH. (Two-way ANOVA, Condition main effect: (F (3, 45) = 7.765, *p* = 0.0003), Brain area main effect: (F (2, 45) = 209.4, *p* < 0.0001), Condition ×Brain area interaction: (F (6, 45) = 4.381, *p* = 0.014), followed by Holm–Sidak post-hoc analysis). (**B**) Photomicrographs (25×, dorsal view) illustrate Hcrt neurons (green) that are located in the aAH and pAH of Control + Control MO (left), Ethanol + Control MO (middle) and Ethanol + *cxcl12a* MO^sp^ (right). Scale bar: 10 µm. All results are shown as means ± standard errors. ** *p* < 0.01, *** *p* < 0.001. Abbreviations: MO: morpholino, aAH: anterior part of the anterior hypothalamus, POA: preoptic area, pAH: posterior part of the anterior hypothalamus, Hcrt: hypocretin, hpf: hours post fertilization, dpf: days post fertilization.

To further test the involvement of Cxcl12a in mediating the effects of embryonic ethanol exposure, we next tested if knockdown of Cxcl12a by injecting a *cxcl12a* MO^sp^ at the single-cell stage blocks the stimulatory effects of ethanol on the number of Hcrt neurons in the aAH and POA. We found as in Experiment 3.2 that the Hcrt neuron number was stimulated in the Ethanol + Control MO zebrafish (Figure 4A), as illustrated in the photomicrographs (Figure 4B). While the Hcrt neurons of the Control + Control MO fish were similar in number in the aAH and pAH and were not evident in the POA, the number of Hcrt neurons in the Ethanol + Control MO fish was increased in the aAH (*p* = 0.0005) but not the pAH (*p* = 0.999), and some (3.2 ± 0.58) ectopically located Hcrt neurons originating in the aAH were observed in the POA (Figure 4A,B). Injection of the *cxcl12a* MO^sp^ in Ethanol + *cxcl12a* MO^sp^ zebrafish blocked the ethanol-induced increase in the number of Hcrt neurons in the aAH (*p* = 0.0012), while having no effect on Hcrt neurons in the pAH (*p* = 0.416). The *cxcl12a* MO^sp^ also completely prevented the formation of ectopic Hcrt neurons further anterior in the POA that were observed in the Ethanol + Control MO zebrafish (Figure 4A,B). Compared to the Control + Control MO zebrafish, injection of the *cxcl12a* MO^sp^ in Control + *cxcl12a* MO^sp^ at the dose used had no effects of its own on the number of Hcrt neurons in the aAH, POA and pAH subpopulations (Table 2). These results provide additional evidence that Cxcl12a is involved in the anatomically specific stimulatory effect of embryonic ethanol exposure on the Hcrt neurons specifically in the aAH and POA subpopulations that is not evident in the pAH.

### 3.5. Cxcl12a Knockdown Blocks the Ethanol-Induced Stimulation of Hcrt Projections to Distant Areas in the Anterior and Posterior Brain

**Figure 5:**
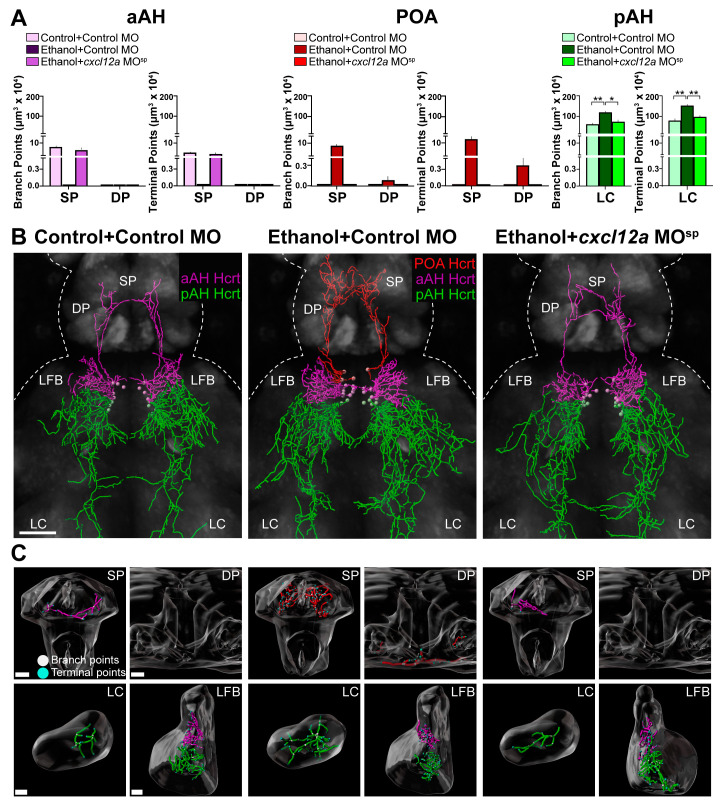
Effects of exposure to embryonic ethanol (0.5% *v*/*v*, 22–24 hpf) and injection of *cxcl12a* MO^sp^ (850 ng/µL) with ethanol on the density of branch points and terminal points of the projections in different brain areas from Hcrt neurons in the aAH, POA and pAH of 6 dpf transgenic *Hcrt:EGFP* zebrafish. (**A**) Bar graphs (n = 4–6/group) with purple-colored bars show projection data of Hcrt neurons located in the aAH and their branch points (left) and terminal points (right) in the SP and DP. Bar graphs (n = 4–6/group) with red-colored bars show projection data of Hcrt neurons located in the POA and their branch points (left) and terminal points (right) in the SP and DP. Bar graphs (n = 4–6/group) with green-colored bars show projection data of Hcrt neurons located in the pAH and their branch points (left, *F* (3, 15) = 4.89, *p* = 0.014) and terminal points (right, *F* (3, 15) = 8.34, *p* = 0.002) in the LC. (**B**) Images show digital representations of Hcrt neuronal projections created from photomicrographs (25×, dorsal view) of Control + Control MO-injected zebrafish (left), Ethanol + Control MO-injected zebrafish (middle) and Control + *cxcl12a* MO^sp^-injected zebrafish (right) obtained using confocal microscopy using the “Filaments” function of Imaris software. The primarily ascending aAH Hcrt projections are shown in purple, the ascending POA Hcrt projections in red and the primarily descending pAH Hcrt projections are shown in green. (**C**) Digitally constructed enlargements of the SP, DP, LC and LFB containing Hcrt projections from the aAH (purple), POA (red) and pAH (green) are shown below, with branch points indicated by white dots and terminal points indicated by blue dots. Scale bars: low magnification 50 µm; SP: 15 µm; DP: 15 µm; LC: 10 µm; LFB: 10 µm. All results are shown as means ± standard errors. * *p* < 0.05, ** *p* < 0.01Abbreviations: aAH: anterior part of the anterior hypothalamus, POA: preoptic area, pAH: posterior part of the anterior hypothalamus, LFB: lateral forebrain bundle, SP: subpallium, DP: dorsal pallium, LC: locus coeruleus, Hcrt: hypocretin, hpf: hours post fertilization, dpf: days post fertilization.

Here we tested whether Cxcl12a knockdown also blocks the stimulatory effects of ethanol on the projections from the Hcrt subpopulations to specific anterior and posterior brain areas. While branch points (3.3 ± 0.45) and terminal points (7.14 ± 1.04) of the long projections from normally located aAH Hcrt neurons in the Control + Control MO zebrafish were evident in the SP but not DP as shown in Experiment 3.3, they were not detected in either area of the Ethanol + Control MO zebrafish (Figure 5A), as illustrated in the photomicrographs (Figure 5B) and brain region enlargements (Figure 5C), again due to the ethanol-induced loss in this group of the most anterior aAH Hcrt neurons with long ascending projections to the SP. Injection of *cxcl12a* MO^sp^ in the ethanol-exposed zebrafish, while preventing the formation of the ectopic POA neurons, restored the branch points (2.56 ± 0.86) and terminal points (5.19 ± 1.48) of the long projections from normally located aAH neurons in the SP, as seen in the Control + Control MO zebrafish (Figure 5A–C). Additionally, in the Ethanol + Control MO zebrafish, the branch points and terminal points of the projections from ectopic POA neurons clearly seen in both the SP (8.64 ± 1.7 and 12.72 ± 2.14, respectively) and DP (0.08 ± 0.05 and 0.37 ± 0.18, respectively) were not detected in the Ethanol + *cxcl12a* MO^sp^ zebrafish similar to the Control + Control MO zebrafish (Figure 5A–C). Like these long anterior projections from POA Hcrt neurons, the Ethanol + Control MO compared to Control + Control MO zebrafish exhibited a significant increase in branch points (*p* = 0.0097) and terminal points (*p* = 0.0014) of the long posterior projections from the pAH Hcrt neurons in the LC. Further, the Ethanol + *cxcl12a* MO^sp^ zebrafish exhibited a blockade of this ethanol effect, showing a significant decrease in the branch points (*p* = 0.025) and terminal points (*p* = 0.0037) of these pAH projections to the LC. Once again, ethanol had no effect on the branch points and terminal points of the short, lateral projections from the aAH and pAH neurons to the LFB (Table 2). In the Control + *cxcl12a* MO^sp^-injected zebrafish compared to the Control + Control MO zebrafish, the *cxcl12a* MO^sp^ had little effect of its own under control conditions, except for reducing the branch points and terminal points of projections in the SP from aAH Hcrt neurons (Table 3). These results, showing that knockdown of Cxcl12a inhibits the ethanol-induced effects on Hcrt projections, further demonstrates that Cxcl12a is directly involved in mediating how ethanol alters the development of the Hcrt neuronal network. 

## 4. Discussion 

This report presents the novel finding that Cxcl12a is directly involved in the effects of embryonic exposure to ethanol on the development of anatomically distinct and ectopically located subpopulations of Hcrt neurons and their projections. As we have recently described [10], this study confirms that embryonic ethanol exposure increases the number of Hcrt neurons specifically in the aAH but not the pAH and causes the most anterior aAH neurons to become ectopically located further anterior in the POA. Notably, overexpression of Cxcl12a closely mimics these stimulatory effects of ethanol on the number of Hcrt neurons normally located in the aAH and abnormally located in the POA, and the knockdown of Cxcl12a via injection of a *cxcl12a* MO^sp^ prevents these specific effects of ethanol. Building on studies in rats showing prenatal Cxcl12 administration to stimulate the overall density of peptide-expressing neurons in the hypothalamus [25] similar to the effects of ventricular injection of Cxcl12 shown in adult rats [41], here we provide the first evidence that embryonic exposure to Cxcl12 can affect specific neuronal subpopulations in a highly localized manner. This agrees with evidence that Cxcr4, the receptor for Cxcl12, is also involved in these anatomically specific effects, with embryonic pretreatment of the Cxcr4 antagonist AMD3100 found to block the ethanol-induced increase in the number of Hcrt neurons in the aAH and induction of ectopic Hcrt neurons in the POA [10]. 

The increase in number of Hcrt neurons in the aAH and POA induced similarly by ethanol and *cxcl12a* mRNA injection may be due, in part, to an increase in their proliferation and migration, which is stimulated by low-moderate concentrations of embryonic ethanol [6,11,42] and by Cxcl12 itself [24,43,44]. With embryonic ethanol exposure shown to increase the expression of Cxcl12 in the hypothalamus [22,23], Cxcl12a overexpression in turn likely increases the proliferation and migratory speed of the aAH Hcrt neurons, specifically those located in the most anterior region of the aAH, causing them to become ectopically expressed in the POA. Importantly, this induction of ectopic neurons in the POA by both ethanol and Cxcl12a overexpression is not simply due to a redistribution of Hcrt neurons from the aAH but occurs in conjunction with an increase in Hcrt number in the aAH. Further evidence linking the ethanol-induced increase in proliferation to Cxcl12 is suggested by our recent studies [9,10], showing that the more anterior areas of the aAH and POA have naturally higher levels of *cxcl12a* transcripts along with greater proliferative activity compared to the posterior area of the pAH. While it is possible that the increased number of Hcrt neurons produced by both ethanol and Cxcl12a overexpression results from decreased programmed cell death of these neurons, prior studies of embryonic exposure at comparably low concentrations of ethanol in both rats and zebrafish have reported no changes in apoptosis [5,45].

Cxcl12 has a well-established role in controlling migration, as shown in rodent studies of neurons expressing gonadotropin-releasing hormone (GnRH) [28,46] and of trigeminal sensory and motor neurons [47,48], consistent with an in vitro study showing the velocity and distance migrated by hypothalamic peptide-expressing neurons to be increased by the chemokine CCL2 [49]. With both Cxcl12a overexpression and ethanol exposure increasing Hcrt neuron number, future studies will be needed to determine if the combination of these two treatments produces an additive effect and an even greater increase in Hcrt number, or if there is a ceiling to the increase in Hcrt neuron number. Further, with Cxcl12a MO^sp^ administration blocking the ethanol-induced increase in Hcrt neurons, future work will be needed to evaluate Cxcl12 as a potential target to reverse the increased recruitment of Hcrt neurons produced by a number of substances of misuse, including cocaine [50] and alcohol [23].

The induction of ectopic Hcrt neurons in the POA from the anterior aAH subpopulation may also involve an endogenous Cxcl12 concentration gradient, where neurons that express Cxcr4 migrate towards the higher concentrations of this chemokine. While the role of a Cxcl12a gradient in cellular migration has been clearly demonstrated in peripheral systems such as the lateral line in zebrafish [51] and the in vitro retinal ganglion cells from mice [52], we recently found in the embryonic zebrafish brain a natural posterior-to-anterior concentration gradient of *cxcl12a* transcripts, with the lowest levels in the pAH, higher levels in the aAH and POA and the highest levels in the telencephalon [10]. We also discovered that ethanol stimulates *cxcl12a* transcript expression along this natural gradient, while having an anatomically specific stimulatory effect on the co-expression of Cxcr4b in Hcrt neurons in the aAH and POA subpopulations but not in the pAH [10]. Similar to ethanol, the injection of *cxcl12a* mRNA likely increases endogenous Cxcl12a expression along this gradient causing Cxcr4b-expressing Hcrt neurons to migrate towards the higher Cxcl12a levels in the anterior brain, while injection of the *cxcl12a* MO^sp^ likely has the opposite effect on Cxcl12a expression, thereby blocking the ethanol-induced abnormal migration of aAH Hcrt neurons further anterior into the POA. This idea is further supported by evidence showing that the transplantation of Cxcl12a in the anterior zebrafish brain causes trigeminal Cxcr4b-expressing sensory neurons to migrate anteriorly towards the transplanted Cxcl12a [53] and by in vitro findings showing dorsal root ganglion neurons to be attracted to the local source of Cxcl12 [54]. Another possible mechanism in the anatomically specific effects of ethanol and Cxcl12a on Hcrt neurons may involve radial glia progenitor cells. These neuroprogenitor cells, which in zebrafish and rats have an important role in cell proliferation and migration [55,56,57], are densely concentrated in the hypothalamus where they co-express chemokines [23,58] and are strongly stimulated by embryonic ethanol exposure, consequently contributing to alterations in the proliferation and migration of Hcrt neurons. 

In addition to controlling neuronal proliferation and migration, Cxcl12 is also known to mediate the development of neuronal projections, including projection pathfinding and positioning, axonal elongation, branch formation and dendrite generation and density [26,27,28,29]. Here we provide the first direct evidence that Cxcl12a mediates the effects of embryonic ethanol exposure on the development of Hcrt neuronal projections, with Cxcl12a overexpression producing stimulatory effects similar to ethanol. It is worth noting, however, that the effects of Cxcl12a overexpression on Hcrt projections are not as strong as those produced by ethanol, including a lack of an effect of Cxcl12a overexpression on DP projections from POA Hcrt neurons that was seen in ethanol-exposed fish. This may be due to Cxcl12a overexpression producing less of an increase in Cxcl12a protein levels, possibly via some mRNA degradation, or due to the involvement of other neuroimmune mediators, such as the CCL2/CCR2 chemokine system, shown to be stimulated by embryonic ethanol and to alter neuronal development [59]. While having no effect on the short, lateral projections of Hcrt neurons to the LFB, Cxcl12a overexpression like ethanol increases the branch points and terminal points of the long projections from the aAH, POA and pAH subpopulations. Although the mechanisms have yet to be determined, this finding that ethanol has differential effects on Hcrt neuronal projections depending on their length is similar to a prior report in mice [60], showing that early exposure to ethanol differentially affects the long and short projections from pyramidal neurons in the medial prefrontal cortex. 

It is notable that the Hcrt neurons, which become ectopically expressed in the POA after ethanol exposure and Cxcl12a overexpression, share the same projection characteristics as the most anterior aAH neurons normally located in control zebrafish, with both having long ascending projections to the SP. This supports the idea that these ethanol-induced ectopic neurons in the POA do, in fact, originate from the anterior aAH subpopulation. Our results, showing that knockdown of Cxcl12a completely blocks the formation of these ectopic POA neurons with long ascending projections and restores their position in the anterior aAH region, provides evidence that Cxcl12a is an important mediator of these ethanol effects on the location of Hcrt neurons. Our findings also suggest that Cxcl12a plays a critical role in the anterior pathfinding of the Hcrt projections, with injection of the *cxcl12a* MO^sp^ decreasing the branch points and terminal points of aAH Hcrt neurons in the SP of control zebrafish and completely blocking the ethanol-induced branch points and terminal points in the DP, a more dorsal region not typically innervated by Hcrt neurons at this young age [36].

With our recent study showing ethanol to increase the concentration gradient of Cxcl12a in the brain with highest levels in the telencephalon where the SP and DP are located [10], it is likely that this gradient also contributes to the ethanol-induced guidance of the Hcrt neuron projections in the anterior direction. With Hcrt soma found to co-express Cxcr4b receptors in both rats and zebrafish [10,23], the Hcrt neuronal projections also likely co-express Cxcr4b receptors that contribute to the guidance of these projections towards higher Cxcl12a concentrations. This idea is consistent with findings in mice, showing that Cxcr4 receptors are expressed along the axons of embryonic GnRH neurons [46] and Cxcl12 is expressed at high levels where motor neuron projections terminate [61], and also in zebrafish, where both Cxcl12a and Cxcr4b expression are positively correlated with the axonal pathway of GnRH projections [28]. In addition to these effects on the anterior Hcrt projections, Cxcl12a overexpression completely reproduces the effects of ethanol on the posterior projections from pAH neurons, increasing their branch points and terminal points in the LC, an effect that is totally blocked by Cxcl12a knockdown in ethanol-exposed fish. Although a chemokine concentration gradient has yet to be described in the area of the LC, there are previous reports showing that Cxcr4 is expressed in the LC of both rats and humans [62,63], indicating that this chemokine system may be associated with the pathfinding of neuronal projections to this region. 

The effects described here of ethanol and Cxcl12a overexpression on the development of different subpopulations of Hcrt neurons and their projections are likely to have significant behavioral consequences. In both zebrafish and rats, Hcrt has a positive relationship with behaviors that are stimulated by embryonic ethanol exposure [36,42], including alcohol consumption [64,65] as well as locomotor activity and anxiety-like behavior [42,66,67]. Similar to ethanol, chemokines are also shown to stimulate these behaviors [25,68], and chemokine receptor antagonists are found to block the behaviors induced by ethanol in larval zebrafish [10] as well as rats [68]. Studies in rodents show that Hcrt neuron subpopulations mediate different behaviors related to substances of misuse [50,69], possibly due to the known neurochemical and genetic heterogeneity of Hcrt neurons [70]. For example, Hcrt neurons co-express other neurochemicals such as dynorphin and glutamate [36,71], and it is possible that embryonic ethanol exposure or Cxcl12a overexpression alters their level of co-expression within specific subpopulations of Hcrt neurons that in turn affect neuronal functioning and contribute to behavioral disturbances. Our laboratory recently demonstrated the functional importance of specific Hcrt subpopulations in mediating ethanol-induced behaviors [7], showing that ablation of the ectopic POA Hcrt neurons or the Hcrt neurons in the most anterior part of the aAH blocks the ethanol-induced increase in anxiety-like behavior, and ablation of the ectopic Hcrt neurons blocks the ethanol-induced increase in locomotor behavior. In this report, we take the next step and reveal that Cxcl12a is involved in mediating the ethanol-induced effects on the projections of different Hcrt neuron subpopulations to functionally different brain areas. Ethanol and Cxcl12a both induce Hcrt neurons in the most anterior region in the aAH that become ectopically expressed in the POA and send long projections to the SP, a brain area homologous to the mammalian basal ganglia that contains Hcrt receptors and is rich in cells expressing neurotransmitters associated with ethanol-related behaviors [72,73,74]. Notably, ethanol also causes ectopic Hcrt neurons in the POA to send projections further anterior to the DP, an area shown to participate in drug responses of zebrafish [75,76]. In contrast, the Hcrt subpopulation in the pAH innervates the LC, a region that is rich in norepinephrine and important for locomotor and sleep–wake behaviors [77,78] and has a functional relationship with Hcrt neurons in terms of these behaviors [79]. These alterations in Hcrt projections within the LC may act as a potential mechanism underlying sleep disorders that are often comorbid with substance use disorders [80], consistent with evidence showing Hcrt receptor antagonists to have clinical efficacy in improving sleep and reducing withdrawal symptoms [81]. Together, our finding here, that Cxcl12a knockdown blocks the ethanol-induced effects on these Hcrt projections from the Hcrt subpopulations to distant brain areas, suggests that this knockdown will also reduce or block the behaviors induced by ethanol exposure. 

In summary, this study demonstrates that the chemokine Cxcl12a directly mediates the stimulatory effects of embryonic ethanol exposure on the number of Hcrt neurons in anatomically specific subpopulations and the induction of ectopic Hcrt neurons, effects mimicked by injection of *cxcl12a* mRNA and blocked by knockdown using a *cxcl12a* MO^sp^. This chemokine is also involved in ethanol’s effects on the pathfinding of projections from Hcrt neurons to distant brain areas known to have important behavioral functions. These neurodevelopmental changes likely alter the functioning of Hcrt neurons, contributing to disturbances in alcohol-related behaviors shown to be produced by embryonic ethanol exposure. 

## Figures and Tables

**Table 1:** Effects of ethanol and *cxcl12a* mRNA injection compared to control on short projections to the lateral forebrain bundle (LFB). One-way ANOVA analysis and post-hoc tests were performed on the density of projection branch points and terminal points of the aAH and pAH neurons innervating the LFB in Ethanol + Vehicle (0.5% *v*/*v*, 22–24 hpf) and Control + *cxcl12a* mRNA (50 ng/µL) zebrafish compared to Control + Vehicle *Hcrt:EGFP* zebrafish (6 dpf).

Hcrt Projection Branch and Terminal Points in the LFB				
		**One-way ANOVA**		
**Brain Areas**	**Measures**	***df***	**F**	***p*** **Value**
aAH	Branch Points	2, 10	3.88	0.05
	Terminal Points	2, 10	2.91	1.00
pAH	Branch Points	2, 10	0.47	0.63
	Terminal Points	2, 10	0.29	0.75
		**Post-Hoc Tests**		
**Brain Areas**	**Measures**	**Control +** **Vehicle**	**Ethanol +** **Vehicle**	***p*** **Value**
aAH	Branch Points	8.23 ± 1.79	11.08 ± 0.74	0.35
	Terminal Points	11.70 ± 1.67	16.02 ± 1.49	0.19
pAH	Branch Points	15.16 ± 2.00	11.88 ± 2.04	0.59
	Terminal Points	21.93 ± 3.78	18.26 ± 2.51	0.71
**Brain Areas**	**Measures**	**Control +** **Vehicle**	**Control +** ***cxcl12a*** **mRNA**	***p*** **Value**
aAH	Branch Points	8.23 ± 1.79	5.85 ± 1.17	0.38
	Terminal Points	11.70 ± 1.67	10.87 ± 0.63	0.67
pAH	Branch Points	15.16 ± 2.00	13.93 ± 3.26	0.93
	Terminal Points	21.93 ± 3.78	19.32 ± 4.35	0.71

Post-hoc data are represented as mean ± SEM. Branch points and terminal points are measured as density (µm^3^ × 10^4^). Abbreviations: Hcrt: Hypocretin, aAH: anterior part of the anterior hypothalamus, POA: preoptic area, pAH: posterior part of the anterior hypothalamus; LFB: lateral forebrain bundle.

**Table 2:** Effects of ethanol and *cxcl12a* MO^sp^ injection compared to control on short projections to the lateral forebrain bundle (LFB). One-way ANOVA analysis and post-hoc tests were performed on the density of branch points and terminal points of the projections from aAH and pAH neurons innervating the LFB in Ethanol + Control MO (0.5% *v*/*v*, 22–24 hpf) zebrafish compared to Control + Control MO and Ethanol + *cxcl12a* MO^sp^ (850 ng/µL) *Hcrt:EGFP* zebrafish (6 dpf).

Hcrt Projection Branch and Terminal Points in the LFB				
		**One-way ANOVA**		
**Brain Areas**	**Measures**	***df***	**F**	***p*** **Value**
aAH	Branch Points	2, 10	3.88	0.05
	Terminal Points	2, 10	2.91	1.00
pAH	Branch Points	2, 10	0.47	0.63
	Terminal Points	2, 10	0.29	0.75
		**Post-Hoc Tests**		
**Brain Areas**	**Measures**	**Control +** **Control MO**	**Ethanol + ** **Control MO**	***p*** **Value**
aAH	Branch Points	10.44 ± 2.99	8.11 ± 1.44	0.97
	Terminal Points	14.59 ± 3.12	11.81 ± 1.83	0.52
pAH	Branch Points	12.21 ± 1.51	10.60 ± 1.83	0.69
	Terminal Points	20.84 ± 1.59	19.03 ± 3.02	0.64
**Brain Areas**	**Measures**	**Ethanol +** **Control MO**	**Ethanol +** ***cxcl12a*** **MO^sp^**	***p*** **Value**
aAH	Branch Points	8.11 ± 1.44	2.99 ± 0.77	0.15
	Terminal Points	11.81 ± 1.83	5.15 ± 0.1.51	0.20
pAH	Branch Points	10.60 ± 1.83	7.68 ± 1.97	0.57
	Terminal Points	19.03 ± 3.02	11.96 ± 2.59	0.16

Post-hoc data are represented as mean ± SEM. Branch points and terminal points are measured as density (µm^3^ × 10^4^). Abbreviations: Hcrt: Hypocretin, aAH: anterior part of the anterior hypothalamus, POA: preoptic area, pAH: posterior part of the anterior hypothalamus; LFB: lateral forebrain bundle.

**Table 3:** Effects of *cxcl12a* MO^sp^ injection in control zebrafish on Hcrt neuron number and projections in brain areas of interest. Two-way ANOVA analysis and post-hoc tests were performed on the number of Hcrt neurons in the aAH, POA and pAH, and *t* test analysis was performed on the density of projection branch points and terminal points of aAH and pAH neurons, with all comparisons made between Control + Control MO and Control + *cxcl12a* MO^sp^ (850 ng/µL) *Hcrt:EGFP* zebrafish (6 dpf).

Hcrt Neuron Number			Two-Way ANOVA Post-Hoc Tests		
**Brain Areas**		**Measures**	**Control +** **Control MO**	**Control +** ***cxcl12a*** **MO^sp^**	***p*** **Value**
aAH		# Hcrt neurons	9.50 ± 0.28	10.00 ± 0.41	0.85
POA		# Hcrt neurons	0	0	NA
pAH		# Hcrt neurons	9.25 ± 0.47	9.25 ± 0.75	0.99
**Hcrt Projection Branch and Terminal Points**			***t*** **test results**		
**Brain Areas**	**Projection Areas**	**Measures**	**Control +** **Control MO**	**Control +** ***cxcl12a*** **MO^sp^**	***p*** **Value**
aAH	SP	Branch Points	3.30 ± 0.45	0.54 ± 0.34	0.003 **
		Terminal Points	7.14 ± 1.03	3.00 ± 1.30	0.047 *
	DP	Branch Points	0	0	NA
		Terminal Points	0	0	NA
	LFB	Branch Points	10.44 ± 2.99	6.05 ± 1.66	0.24
		Terminal Points	14.59 ± 3.12	16.01 ± 1.52	0.19
POA	SP	Branch Points	0	0	NA
		Terminal Points	0	0	NA
	DP	Branch Points	0	0	NA
		Terminal Points	0	0	NA
pAH	LC	Branch Points	60.52 ± 1.51	92.43 ± 23.18	0.32
		Terminal Points	80.98 ± 8.95	111.24 ± 24.73	0.16
	LFB	Branch Points	12.21 ± 1.51	8.50 ± 1.50	0.13
		Terminal Points	20.84 ± 1.59	14.77 ± 2.51	0.08

Significant effects are boldface. * *p* < 0.05; ** *p* < 0.01. Post-hoc data are represented as mean ± SEM. Branch points and terminal points are measured as density (µm^3^ × 10^4^). Abbreviations: Hcrt: Hypocretin, MO: morpholino; MO^sp^: splice morpholino, #: number; aAH: anterior part of the anterior hypothalamus, POA: preoptic area, pAH: posterior part of the anterior hypothalamus, SP: subpallium; DP: dorsal pallium; LFB: lateral forebrain bundle; LC locus coeruleus.

## Data Availability

The datasets used and/or analyzed during the current study are available from the corresponding author on reasonable request.
